# Pro-phagocytic function and structural basis of GPR84 signaling

**DOI:** 10.21203/rs.3.rs-2535247/v1

**Published:** 2023-02-15

**Authors:** Xuan Zhang, Yujing Wang, Shreyas Supekar, Xu Cao, Jingkai Zhou, Jessica Dang, Siqi Chen, Laura Jenkins, Sara Marsango, Xiu Li, Guibing Liu, Graeme Milligan, Mingye Feng, Hao Fan, Weimin Gong, Cheng Zhang

**Affiliations:** 1 Division of Life Sciences and Medicine, University of Science and Technology of China, Hefei, Anhui, China.; 2 Department of Pharmacology and Chemical Biology, University of Pittsburgh School of Medicine, University of Pittsburgh, Pittsburgh, PA 15261, USA.; 3 Bioinformatics Institute (BII), Agency for Science, Technology and Research (A*STAR), 138671, Singapore.; 4 Department of Immuno-Oncology, Beckman Research Institute, City of Hope Comprehensive Cancer Center, Duarte, CA 91010, USA.; 5 Centre for Translational Pharmacology, School of Molecular Biosciences, College of Medical, Veterinary and Life Sciences, University of Glasgow, Glasgow G12 8QQ, Scotland, U.K.

## Abstract

GPR84 is a unique orphan G protein-coupled receptor (GPCR) that can be activated by endogenous medium-chain fatty acids (MCFAs). The signaling of GPR84 is largely pro-inflammatory, which can augment inflammatory response, and GPR84 also functions as a pro-phagocytic receptor to enhance phagocytic activities of macrophages. In this study, we first showed that the activation of GPR84 by the synthetic agonist 6-OAU could synergize with the blockade of CD47 on cancer cells to induce phagocytosis of cancer cells by macrophages. Then, we determined a high-resolution structure of the GPR84-G_i_ signaling complex with 6-OAU. This structure revealed a completely occluded binding pocket for 6-OAU, the molecular basis of receptor activation involving non-conserved structural motifs of GPR84, and an unusual G_i-_coupling interface. Together with computational docking and simulations studies, our structure also suggested the mechanism for the high selectivity of GPR84 for MCFAs and the potential routes of ligand binding and dissociation. Our results provide a framework for understanding GPR84 signaling and developing new drugs targeting GPR84.

## INTRODUCTION

Free fatty acids (FFAs) are a unique group of lipid species, derived from triglycerides upon lipolysis. They can signal through a group of G protein-coupled receptors (GPCRs)^1,2^ to function in metabolism, inflammation and immunity^3–6^. GPR84 is a G_i_-coupled GPCR that has been suggested to recognize endogenous medium-chain fatty acids (MCFAs) but not short- or long-chain fatty acids (SCFAs and LCFAs)^7^ (**Fig. S1**). Among native fatty acids, capric acid with a 10-carbon atom chain length showed the highest potency for activating GPR84^7^. Nevertheless, the low potency of those lipids and the lack of evidence suggesting the involvement of GPR84 in the physiological function of MCFAs obscures their exclusive physiological pairing with the receptor^8^. Therefore, GPR84 still remains as an orphan GPCR. Nevertheless, GPR84 was found to be predominantly expressed by immune cells^7–9^, and its expression can be strongly up-regulated under inflammatory conditions to augment inflammatory responses and enhance phagocytosis^10–13^. Using synthetic GPR84 agonists and antagonists as useful pharmacological tools, previous research revealed the pro-inflammatory function of GPR84 signaling in various pathological conditions^11,13–15^. In particular, GPR84 signaling has been shown to promote fibrosis^15,16^. Several GPR84 antagonists were developed for therapeutic purposes. Two of them, PBI-4050 and GLPG1205, have been tested in clinical trials for treating pulmonary fibrosis^17–20^, although no significant therapeutic efficacy was reported so far.

One of the immunological functions of GPR84 signaling is to promote macrophage phagocytosis^10,21^. This has been indicated in a recent study for cancer cells^12^. This study identified an enzyme expressed in cancer cells named APMAP (Adipocyte Plasma Membrane Associated Protein) that functions as an anti-phagocytic factor to impede antibody-dependent cellular phagocytosis (ADCP) of cancer cells induced by blocking CD20^12^. Loss of the *APMAP* gene can significantly enhance the macrophage phagocytosis of cancer cells, which is dependent on GPR84 and G_i_^12^. Analysis of previous RNA-sequencing data of human tumors also suggested specific expression of GPR84 in tumor-associated macrophages (TAMs)^12^. All the data suggested a critical role of the GPR84-G_i_ signaling axis in mediating phagocytic activities of macrophages especially TAMs against cancer cells.

A major breakthrough in cancer immunosurveillance was the identification of ‘don’t eat me’ signals such as CD47, which can be upregulated on cancer cells to inhibit macrophage phagocytosis^22,23^. Blocking the interaction between such signals and their macrophage-expressing receptors triggers cancer cell phagocytosis, leading to promising anticancer effects in mouse cancer models and clinical trials^22,23^. To further explore the therapeutic potential of activating GPR84 signaling in cancer, we first proved that activation of the GPR84-G_i_ signaling axis by the commonly used synthetic GPR84 agonist 6-OAU (6-n-octylaminouracil)^7,11^ could synergize with an anti-CD47 antibody^24,25^ that disrupts the binding of CD47 to its receptor, Sirpa^23^, on macrophages to induce phagocytosis of cancer cells by macrophages. To understand the actions of 6-OAU at a molecular level and to facilitate the potential rational development of other, more drug-like, GPR84 activators, we then determined a high-resolution cryo-electron microscopy (cryo-EM) structure of the GPR84-G_i_ signaling complex with 6-OAU. Our structure revealed a completely occluded binding pocket for 6-OAU and a receptor-specific Gi-coupling mode. Together with computational docking and simulations studies, our structure provides unprecedented insights into the lipid recognition by GPR84 and the receptor activation mechanism. We expect that our results will facilitate future drug development on GPR84 for cancer and other inflammatory diseases.

## RESULTS

### Pro-phagocytic effect of GPR84-Gi signaling in cancer cell phagocytosis by macrophages

**Figure 1.**
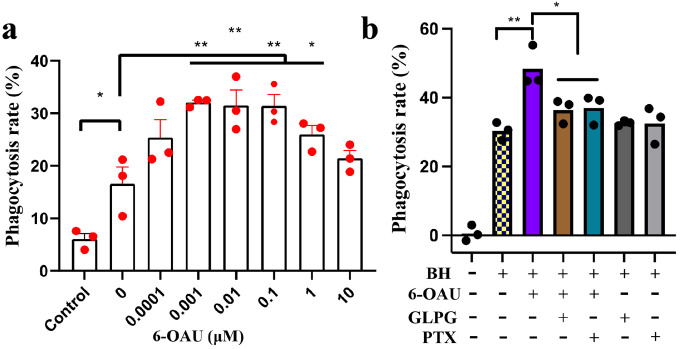
GPR84-Gi signaling facilitates cancer cell phagocytosis. **(a)** Dose-dependent pro-phagocytic effect of 6-OAU. **(b)** GLPG1205 and pertussis toxin (PTX) abolished the pro-phagocytic effect of 6-OAU. BH means B6H12, the CD47 blocking antibody. PTX means pertussis toxin. Each data point represents SD of data from 3 independent experiments (n=3) taken from distinct samples. *p<0.05 and **p<0.01.

Previous studies showed that GPR84 agonists could enhance the antibody-dependent cellular phagocytosis (ADCP) of B lymphocytes in the presence of an anti-CD20 antibody^12^. Here, we further tested the effect of 6-OAU with the CD47-blocking antibody, B6H12, in cancer cell phagocytosis by bone marrow-derived macrophages (BMDMs). Circulating monocytes that originate from bone marrow are constantly recruited to tumor sites and develop into TAMs. Therefore, BMDMs have been established as a sound model for studying phagocytosis of tumor cells. We used BMDMs from BALB/c mice whose Sirpa displays a binding affinity to human CD47 comparable to that of human Sirpa^26,27^. Our results indicated that treatment of BMDMs with 6-OAU promoted the phagocytosis of Raji cells, a human non-Hodgkin lymphoma cell line, in a concentration-dependent manner (Fig. 1a). To prove this effect was GPR84 dependent, we used the GPR84-specific antagonist GLPG1205^28^ and showed that blocking GPR84 activation with GLPG1205 completely abolished the pro-phagocytic effect of 6-OAU (Fig. 1b). In addition, this effect of 6-OAU was also abolished by pre-treatment with the G_i_ protein blocker pertussis toxin, confirming that the pro-phagocytic action of GPR84 is dependent on the G_i_ signaling (Fig. 1b). Altogether, our data suggested that activation of the GPR84-G_i_ signaling axis in macrophages can synergize with CD47 blockade to drive the phagocytosis of cancer cells.

### Structure of the 6-OAU-GPR84-Gi complex and an occluded ligand binding pocket

**Figure 2.**
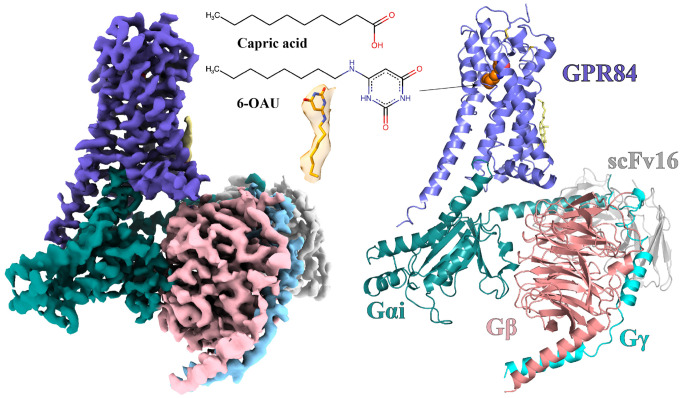
Overall structure of the 6-OAU-GPR84-Gi complex. The left and right panels show the cryo-EM density map and the overall structure, respectively. The chemical structures of capric acid and 6-OAU and the cryo-EM density are shown in the middle. GPR84 is colored in blue. Gαi, Gβ and Gγ subunits are colored in cyan, pink and light blue, respectively. ScFv16 is colored in grey.

To understand how 6-OAU activates the GPR84-G_i_ signaling axis, we sought to determine a high-resolution structure of the 6-OAU-GPR84-G_i_ complex by cryo-EM. We assembled the complex using the NanoBit tethering strategy in insect Sf9 cells^29^. The complex was treated with apyrase to hydrolyze GDP to ensure the α subunit of G_i_, G_αi_, remained in a nucleotide-free state^30^. An antibody fragment, scFv16, was used to stabilize the G_i_ heterotrimer^31^. The structure was determined to a global resolution of 3.0-Å by cryo-EM (Fig. 2, **Figs. S2, Table S1**). The clear cryo-EM density of the receptor allowed us to model the residues from D6 to P389 of GPR84 except for the long intracellular loop 3 (ICL3) from L217 to F314 in the structure. For the heterotrimeric G_i_ protein, the helical domain of G_αi_ was not modeled due to potential structural flexibility^32^.

**Figure 3.**
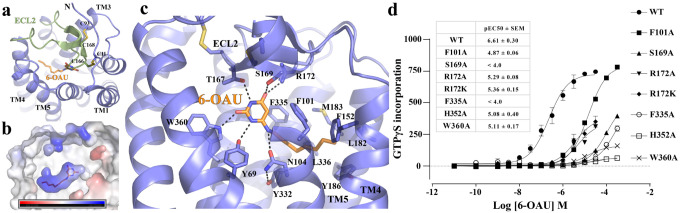
6-OAU binding in GPR84. **(a)** Occluded binding pocket for 6-OAU covered by ECL2. **(b)** Charge potential of the 6-OAU binding pocket. The bar shows the levels of negative (red) and positive (blue) charge potential. **(c)** Interactions between 6-OAU and GPR84. The polar interactions are shown as dashed lines. **(d)** Mutagenesis data using GTPγS incorporation assays. Data represent mean ± SEM from at least 3 independent experiments. 6-OAU is shown as orange sticks in all figures.

The overall structure of GPR84 resembles those of other Class A rhodopsin-like GPCRs^33^. The extracellular loop 2 (ECL2), which is almost perpendicular to the 7-transmembrane helical bundle (7-TM), adopts a β-hairpin structure to extend towards transmembrane helix 1 (TM1) on top of the 6-OAU binding pocket, shielding it from the extracellular milieu (Fig. 3a). Two disulfide bonds further stabilize the conformation of ECL2; One forms between C168 of ECL2 and C93^3.25^ (superscripts represent Ballesteros-Weinstein numbering^34^) of TM3, which is highly conserved in Class A GPCRs^35^, and the other forms between C166 of ECL2 and the N-terminal residue C11. The latter has also been proposed in a previous modeling study^36^. In addition, no openings between transmembrane helices are observed around 6-OAU. As a result, the ligand is completely buried inside the 7-TM and occluded from the outside aqueous and lipidic environment (Fig. 3b). A similar completely occluded ligand-binding pocket has also been observed in another lipid GPCR, the cannabinoid receptor 2 (CB2)^37,38^ (**Fig. S3)**. However, different from GPR84, in the structure of active CB2 with G_i_, a part of the N-terminal region of CB2 folds on top of the ligand binding pocket to shield it from the extracellular environment^37,38^ (**Fig. S3)**.

6-OAU is an amphipathic molecule with a polar head group and an octylamine tail (Fig. 1). Accordingly, multiple polar and hydrophobic interactions between 6-OAU and GPR84 are observed (Fig. 3c, **Fig. S4**). The uracil head group of 6-OAU engage in extensive hydrogen-bonding interactions with T167, S169 and R172 in ECL2 and Y69^2.53^ and W360^7.43^ of GPR84. The amine group of the octylamine tail of 6-OAU also forms a salt bridge with N104^3.36^. The mutation of T167A has been shown to abolish the action of capric acid^39^. We also found that mutations of S169A, W360A, and R172A could make the receptor much less responsive to 6-OAU (Fig. 3d), proving the important roles of the polar interactions with GPR84 in the agonistic action of 6-OAU. Interestingly, R172K caused an even more significant change of the EC_50_ of 6-OAU than that caused by R172A. It is possible that R172K may result in new interactions and cause conformational changes of ECL2 to disrupt 6-OAU binding. In addition to the polar interactions, the saturated octyl tail of 6-OAU resides in a hydrophobic sub-pocket surrounded by GPR84 residues F101^3.33^, F152^4.57^, L182^5.42^, Y186^5.46^, Y332^6.48^, F335^6.51^, L336^6.52^, and L361^7.44^ (Fig. 3c, **Fig. S4)**. Consistent with such finding, our mutagenesis studies showed that F101A and F335A resulted in much-compromised action of 6-OAU (Fig. 3d).

**Figure 4.**
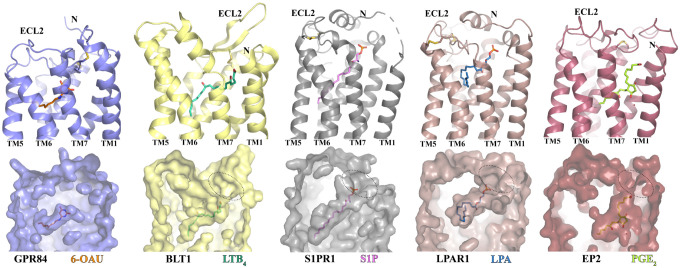
Comparison of the ligand binding pockets in GPR84 and four other lipid GPCRs. BLT1, S1PR1, LPAR1, and EP2 are receptors of LTB_4_, S1P, LPA, and PGE_2_, respectively. The structures of GPR84, BLT1 (PDB ID 7VKT), S1PR1 (PDB ID 7TD3), LPAR1 (PDB ID 7TD0), and EP2 (PDB ID 7CX2) are colored slate, light yellow, grey, brown, and dark red, respectively. All ligands are shown in sticks.

The overall binding pose of 6-OAU is similar to those of leukotriene B_4_ (LTB_4_)^40^, sphingosine 1-phosphate (S1P)^41–44^, lysophosphatidic acid (LPA)^45^, and prostaglandin E2 (PGE_2_)^46^ in their respective GPCRs (Fig. 4). In the structures of these four lipids with their receptors, the carboxylate head group of each lipid is located near the extracellular surface while the hydrophobic carbon chains are buried inside the 7-TM bundle (Fig. 4). The binding pockets of all four lipids have openings at the extracellular regions of their respective receptors, potentially serving as the ligand entrance (Fig. 4). This is in contrast to the occluded binding pocket of 6-OAU. Also, in GPR84, ECL2 inserts into the 7-TM region, resulting in a much shorter binding pocket compared to those in the receptors for LTB_4_, S1P, LPA, and PGE_2_ (Fig. 4), explaining why GPR84 doesn’t bind to LCFAs^7^. Analysis of the charge potential of the 6-OAU binding pocket showed an uneven positive charge distribution (Fig. 3b). A similar uneven distribution of the positive charge potential was observed for the ligand binding pocket in the prostaglandin D_2_ (PGD_2_) receptor DP2, which has been proposed to facilitate the recognition of PGD_2_ by DP2^47,48^. For GPR84, previous studies suggested that the positive charge of R172 in the ECL2 plays a key role in the binding of MCFAs by coordinating the carboxylate head group^49,50^.

### Ligand recognition mechanisms revealed by computational docking and MD simulations

**Figure 5.**
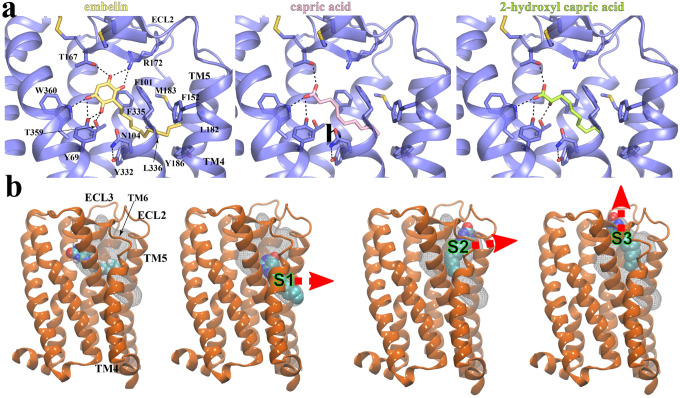
Docking of GPR84 agonists and MD simulations of 6-OAU-bound GPR84. **(a)** Interactions of docked embelin (light yellow), capric acid (pink), and 2-hydroxyl capric acid (lime) with GPR84 (slate). Hydrogen bonds are shown as black dashed lines. **(b)** Exit routes of 6-OAU in MD simulations. The 6-OAU movement during the simulations is shown as density in white grid. Red arrows indicate possible ligand exit routes via metastable sites S1, S2 or S3. 6-OAU is shown as cyan spheres and GPR84 is shown in orange.

To further investigate how GPR84 recognizes different agonists, we sought to dock three other GPR84 agonists, embelin, capric acid, and 2-hydroxy capric acid, to the GPR84 structure. To validate our docking methods, we first docked 6-OAU to our structure, which recapitulated the 6-OAU binding pose observed in our structure with slight differences at the lipid tail, implying a high flexibility of this part (**Fig. S5a**). Our docking results showed that embelin, capric acid, and 2-hydroxy capric acid adopt similar binding poses as 6-OAU (**Fig. S5b**), in which their polar groups located near ECL2 engage in different sets of hydrogen-bonding interactions with nearby GPR84 residues and their lipid tails stick into the same hydrophobic pocket towards the cleft between TM4 and TM5 (Fig. 5a). GPR84 residues T167 in ECL2, Y69^2.53^, and W360^7.43^ are involved in the hydrogen boding interactions with all four agonists (Fig. 3c and 5a). The docking scores for these four agonists (**Table S2**) suggest the ranking of their affinities as the following: 6-OAU > embelin > capric acid ≈ 2-hydroxy capric acid, which is in line with their reported EC_50_ values in the literature^8,51^.

To investigate the ligand binding process, we performed large-scale (*ca*. 20 μs) molecular dynamics (MD) simulations of GPR84 in apo (GPR84 alone) and holo (GPR84 with 6-OAU) states. In the holo state simulations, we observed that 6-OAU primarily occupies the native binding pocket (Fig. 5b). However, we found that in several instances 6-OAU indeed moved away from the native state to occupy other metastable sites on the periphery of GPR84 (Fig. 5b, **Fig. S6a**). The first metastable site, namely, site 1 (S1), was located at the interface among TM4-TM5 and membrane lipids (Fig. 5b), where 6-OAU made hydrophobic contacts with membrane lipids and GPR84 residues (**Fig. S6b**). The second metastable site, namely, site 2 (S2), was located at the interface at the TM5-TM6 interface (Fig. 5b), where 6-OAU made H-bonds with membrane lipid headgroups and hydrophobic contacts with GPR84 (**Fig. S6c**). The third metastable site, namely, site 3 (S3), was located on top of the orthosteric site near ECL2-ECL3-water interface (Fig. 5b). At site 3, R172 at the base of ECL2 β-hairpin made a cation-π interaction with 6-OAU, presumably acting as a gatekeeper residue preventing 6-OAU to escape to the solution phase (**Fig. S6d**). The identified peripheral sites suggested putative routes for 6-OAU to exit from the orthosteric site via sites 1 or 2 to the membrane phase, or via site 3 to the extracellular milieu (Fig. 5b, **Fig. S6a**)^52^.

### Non-conserved structural motifs of GPR84 and receptor activation

**Figure 6.**
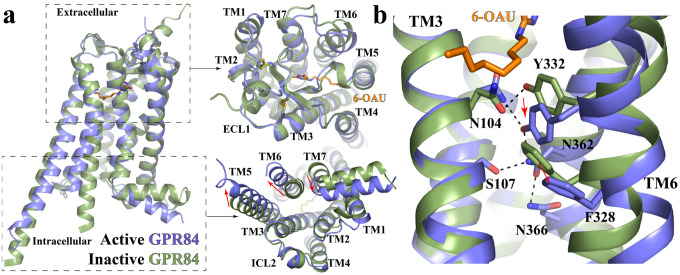
Active conformation of GPR84. **(a)** Superimposition of the active GPR84 structure (blue) to the Alphafold predicted inactive GPR84 structure (green). The extracellular and intracellular regions are shown in the left upper and lower panels, respectively. The red arrows indicate conformational changes of TMs. **(b)** Residues involved in the receptor activation at the core region of GPR84.

Since there is no experimentally solved inactive structure of GPR84, we used the Alphafold predicted structure of apo GPR84^53,54^ in our structural comparison analysis. This structure is expected to represent an inactive conformation since there is no agonist or G protein in the structure. Indeed, structural alignment indicated large conformational rearrangements at the cytoplasmic region including a large outward displacement of TM6 and an inward movement of TM7 of the active GPR84 compared to the Alphafold predicted structure (Fig. 6a). These features are characteristic of receptor activation for Class A GPCRs^55^. In contrast, the extracellular region of GPR84 only showed subtle differences between these two structures (Fig. 6a). It is to be noted that Alphafold successfully predicted the unusual conformation of ECL2 of GPR84 (Fig. 5a)^36^.

For Class A GPCRs, conserved residues W^6.48^ and F^6.44^ form a ‘transmission switch’ motif that connects the extracellular agonist-binding events to the conformational changes at the cytoplasmic regions during receptor activation^56,57^. In GPR84, while F^6.44^ is conserved, W^6.48^ is replaced by a tyrosine residue, Y332^6.48^, which forms a hydrogen bond with N104^3.36^ (Fig. 6b). In the Alphafold predicted structure, Y332^6.48^ also forms hydrogen bonds with N104^3.36^ (Fig. 6b). Structural alignment with the active GPR84 structure showed large rearrangements of these two residues due to the steric effects caused by the octyl tail of 6-OAU (Fig. 6b). It is likely that 6-OAU activates GPR84 mainly by inducing conformational changes of the Y332^6.48^-N104^3.36^ pair, which in turn induce significant displacements of F328^6.44^ and the cytoplasmic segment of TM6 (Fig. 6b). The conformational change of Y332^6.48^ also causes the swing of the side chain of the TM7 residue N362^7.45^. This further results in the formation of a hydrogen bonding network mediated by N362^7.45^ and surrounding residues S107^3.39^, Y332^6.48^, and N366^7.49^ (Fig. 6b), potentially leading to the inward movement of TM7 for G_i_-coupling (Fig. 6b). Such a network is missing in the Alphafold predicted structure (**Fig. S7a**). In addition, N366^7.49^ is a part of the conserved N^7.49^P^7.50^xxY motif^57–59^. This residue forms a salt bridge with D66^2.50^ in the Alphafold predicted inactive structure (**Fig. S7a**). Both residues have been shown to coordinate with a sodium ion in the inactive structures of many other Class A GPCRs, and collapse of this sodium coordination site is involved in the receptor activation^60,61^. Indeed, in the active structure of GPR84, N366^7.49^ moves away from D66^2.50^, which may result from the conformational changes of TM7 in receptor activation.

Another highly conserved structural motif of Class A GPCRs that is not conserved in GPR84 is the D/E^3.49^R^3.50^Y motif. This motif is located near the cytoplasmic surface that mediates intrahelical interactions believed to stabilize the inactive conformation of receptors or modulate receptor activation and G protein coupling^62,63^, which is replaced by G117^3.49^R^3.50^Y in GPR84 (**Fig. S7b**). Two phenylalanine residues F128 and F132 in the intracellular loop 2 (ICL2) and F55^2.39^ in TM2 are in the close vicinity of G117^3.49^ (**Fig. S7b**). They would cause steric clashes if G117^3.49^ is replaced by a glutamic (E) or aspartic (D) acid residue. Interestingly, F128 and F132 in ICL2 form a hydrophobic cluster with F55^2.39^ in TM2 and L121^3.53^ in TM3, potentially stabilizing the α-helical structure of ICL2 (**Fig. S7b**). Such a helical structure of ICL2 is also present in the Alphafold predicted inactive structure of GPR84 (Fig. 6a). This is in contrast to the loop structure of ICL2 in many other Class A GPCRs in the inactive conformation^64^.

### G_i_ coupling mode

**Figure 7.**
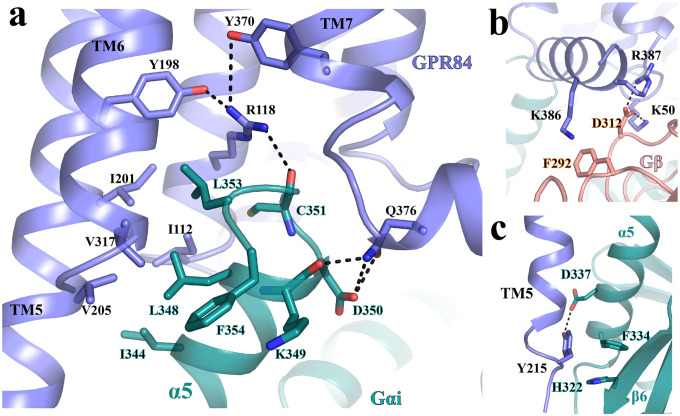
Gi-coupling to GPR84. **(a)** Interactions between GPR84 (blue) and the α5 of G_αi_ (cyan). **(b)** Interactions between GPR84 (blue) and G_β_ (salmon). **(c)** Interactions between the C-terminal end of TM5 of GPR84 (blue) and G_αi_ (cyan). All polar interactions are shown as dashed lines.

In the structure of 6-OAU-bound GPR84-G_i_ complex, G_i_ couples to GPR84 in a canonical way similar to that in the structures of other G_i_-coupled GPCRs. The C-terminal α-helix, α5, of G_αi_ is the major interaction site for GPR84 (**Fig. S8a**). In the C-terminal half of α5 of G_αi_, residues I344, L348, and L353 in the α5 and the last residue, F354, of G_αi_ form hydrophobic interactions with I122^3.54^, I201^5.62^, V205^5.66^, and V317^6.33^ of GPR84 (Fig. 7a). R118^3.50^ of GPR84 in the non-conserved GR^3.50^Y motif mediates a hydrogen-bonding interaction network by interacting with Y198^5.59^ and Y370^7.53^ of GPR84 and with the main-chain carbonyl of C351 of G_αi_, while Q376 in the helix 8 of GPR84 forms hydrogen bonds with the main chain carbonyl of K349 and the side chain of D350 of G_i_ (Fig. 7a). The G_β_ subunit of G_i_ is also involved in direct interactions with GPR84. D312 of G_β_ forms salt bridges with K50 and R387 from ICL1 and helix 8, respectively, of GPR84, and K386 from helix 8 of GPR84 forms a cation-π interaction with F292 of G_β_ (Fig. 7b).

There are some unique features of interactions with G_i_ observed for GPR84. First, the TM5 is much longer than any of the other TMs of GPR84 (Fig. 2). As a result, Y215^5.76^ at the C-terminal end of TM5 of GPR84 forms aromatic and polar interactions with residues F334 and D337 in the C-terminal half of α5of G_αi,_ respectively (Fig. 7c). Another residue, H322, in the β-strand β6 of G_αi_ is also involved in π-π interactions with Y215^5.76^ of GPR84 (Fig. 7c). All of those interactions may facilitate the displacement of α5 of G_αi_, which is translated to the conformational changes of the β6-α5 loop and the release of GDP in G_i_ activation^65^ (**Fig. S8b**). Second, in most of other G_i-_coupled GPCR structures, the position 34.51 in the ICL2 is usually a hydrophobic residue that forms hydrophobic interactions with residues L194 and F336 in Gα_i_. In GPR84, this position is K126. As a result, there is no direct interactions between ICL2 of GPR84 and G_αi._

### Discussion

Our results offer insight into the ligand recognition mechanism for GPR84. First, in our structure, the conformation of ECL2 results in a ligand binding pocket with a size that cannot accommodate LCFAs with 14 or more carbons. In addition, for a potential fatty acid agonist of GPR84, the lipid moiety needs to reach to the bottom region of the binding pocket in order to cause conformational changes of residues including Y332^6.48^ at the core region to activate the receptor. Therefore, the unique shape and size of the binding pocket of GPR84 well explain the preference of the receptor for MCFAs over LCFAs or SCFAs. Second, the occluded binding pocket for 6-OAU makes it difficult to propose a ligand entrance in GPR84. Our MD simulations results suggested three possible routes for 6-OAU to exit the receptor, all of which require conformational changes of the 7-TM region or the extracellular loop region. Interestingly, in the Alphafold predicted inactive structure of GPR84, there are small openings at the extracellular surface between ECL2 and ECL3 and the helical surface between TM5 and TM6 (**Fig. S9**), resembling the S3 and S2 metastable sites in our MD simulations (Fig. 5b and **Fig. S6**). It is likely that the extracellular region of TM5 or TM6 undergoes conformational changes to result in the S2 or S3 site serving as the ligand entrance for the endogenous and synthetic GPR84 ligands.

Tissue macrophages use multiple phagocytic receptors including several opsonic receptors, pattern-recognition receptors (PRRs) and receptor tyrosine kinases (RTKs) to initiate the process of phagocytosis against pathogens (foreign) and apoptotic cells (self)^66,67^. Previous studies^10,12^ and ours suggested that GPR84 serves as a new phagocytic receptor in inflammatory conditions. In particular, the ability of the GPR84 agonist 6-OAU to promote phagocytosis of cancer cells induced by CD47 blockage and the specific expression of GPR84 in TAMs^12^ implied a potential role of GPR84 in cancer immune surveillance. Furthermore, we demonstrated that the G_i_ signaling pathway is critical in the phagocytic function of GPR84 against cancer cells. G_i_ pathway-selective GPR84 agonists, or G_i_-biased GPR84 agonists, may offer a novel therapeutic method to enhance the phagocytosis of cancer cells by macrophages. It has been shown that GPR84 agonists such as 6-OAU could effectively recruit β-arrestins^21,50^, the classic scaffold proteins promoting GPCR internalization and desensitization^68^. Indeed, in our assays, high concentration of 6-OAU led to lowered levels of phagocytosis (Fig. 1a), which was likely due to GPR84 desensitization^69^. In this regard, selective activation of the GPR84-G_i_ pathway with minimal β-arrestin recruitment by G_i-_biased GPR84 agonists such as DL-175^21^ or PSB-16671^50^ may promote more sustained macrophage phagocytosis of cancer cells compared to 6-OAU. In addition, the membrane embedded enzyme APMAP that is highly expressed on the surface of cancer cells has been proposed to degrade the physiological lipid ligand of GPR84 to negatively regulate macrophage phagocytosis^12^. Identifying such a ligand of GPR84 and APMAP may lead to the identification of a novel pathway regulating macrophage function and facilitate the development of novel therapeutics targeting this pathway in addition to GPR84 activators to enhance cancer cell phagocytosis.

## METHODS

### Macrophage phagocytosis assay

The phagocytic ability of macrophages toward live cancer cells was evaluated by a luminescence-based long-term phagocytosis assay as we previously described^70^. Specifically, luciferase-expressing Raji cells were co-cultured with bone marrow-derived macrophages (BMDMs) for 24 h in the absence or presence of CD47 blocking antibody (clone B6H12). Thereafter, the luminescence signal was measured by the addition of luciferin and detection with Cytation 3. For evaluating the effects of GPR84 agonists and/or antagonists, BMDMs were pretreated with corresponding chemicals overnight. After a thorough wash with PBS, the pretreated BMDMs were then used for phagocytosis assay. Cancer cells cultured without BMDMs were used as a normalization control for calculation which indicates a phagocytosis rate of 0%. 6-OAU (0.1 uM) was used to stimulate the activity of GPR84, while GLPG1205 (10 uM) or pertussis toxin (0.1 mg/ml) were used to block the stimulative effect of 6-OUA.

### Protein complex expression and purification

The wild-type human GPR84 was synthesized and cloned into pFastBac vector containing a bovine prolactin signal peptide followed by Flag-tag and His_8_-tag at the N terminus. A fragment of engineered b_2_-adrenergic receptor N-terminal tail region (BN3) was fused GPR84 receptor at the N-terminal end to facilitate protein expression. To enhance the stability of the complex, the NanoBiT tethering strategy was used by fusing a LgBiT subunit at the C-terminus of the receptor^29^. The C-terminal residues G388-H396 was truncated and LgBiT was fused with a 15-amino acid linker (GSSGGGGSGGGGSSG). A dominant negative human Gα_i1_ (DNGα_i1_) containing four mutations (S47N, G203A, E245A, A326S) was cloned into the pFastBac vector^71^. Human Gβ_1_ was fused with an N-terminal His_6_-tag and a C-terminal HiBiT subunit connected with a 15-amino acid linker, was cloned into pFastBac dual vector together with human Gγ_2_.

The expression and purification of scFv16 were achieved as previously described^72^. In brief, the scFv16 was expressed in High Five cells and purified by nickel affinity chromatography before the C-terminal His_8_-tag was removed by TEV protease. The protein was further purified by size exclusion chromatography using a Superdex 200 Increase 100/300 GL column (GE Healthcare). The monomeric peak fractions were pooled, concentrated and stored at −80 °C until use.

GPR84, DNGα_i1_ and Gβ_1_γ_2_ were co-expressed in Sf9 insect cells using Bac-to-Bac baculovirus expression system. Cells were infected with three types of viruses prepared above at the ratio of 1:1:1. After infection for 48 h at 27 °C, cell pellets were harvested and stored at −80 °C until use. Cell pellets were thawed in lysis buffer containing 20 mM HEPES, pH7.5, 50 mM NaCl, 10 mM MgCl_2_, 5 mM CaCl_2,_ 2.5 μg/ml leupeptin, 300 μg/ml benzamidine. To facilitate complex formation, 10 μM 6-OAU, 25 mU/ml Apyrase (NEB), and 100 μM TCEP was added and incubated at room temperature for 2 h. The cell membranes were isolated by centrifugation at 30,700 g for 30 min and then resuspended in solubilization buffer containing 20 mM HEPES, pH7.5, 100 mM NaCl, 0.5% (w/v) lauryl maltose neopentylglycol (LMNG, Anatrace), 0.1% (w/v) cholesteryl hemisuccinate (CHS, Anatrace), 10% (v/v) glycerol, 10 mM MgCl_2_, 5 mM CaCl_2_, 12.5 mU/ml Apyrase, 10 μM 6-OAU, 2.5 μg/ml leupeptin, 300 μg/ml benzamidine, 100 μM TECP for 2h at 4 °C. Insoluble material was removed by centrifugation at 38,900 g for 45 min, and the supernatant was incubated with Ni resin at 4 °C for 2h. The resin was washed with a buffer A containing 20 mM HEPES, pH 7.5, 100 mM NaCl, 0.05% (w/v) LMNG, 0.01% (w/v) CHS, 20 mM imidazole, and 10 μM 6-OAU, 2.5 μg/ml leupeptin, 300 μg/ml benzamidine, 100 μM TECP. The complex was eluted with buffer A containing 400 mM imidazole. The eluate was supplemented with 2mM CaCl_2_ and incubated with an anti-Flag M1 antibody resin overnight at 4 °C. Complex loaded on the Flag column was washed with 10 column volumes of buffer A supplemented 2mM CaCl_2_. Then the complex was eluted by 3.5 column volumes of buffer A containing 5 mM EDTA and 200 μg/ml FLAG peptide. The complex was collected and concentrated using 100 kDa molecular weight cut-off concentrators (Millipore). Purified scFv16 was mixed with eluate at a 1.3:1 molar ratio. The sample was then loaded onto a Superdex 200 Increase 10/300 column (GE Healthcare) pre-equilibrated with buffer containing 20 mM HEPES pH 7.5, 100 mM NaCl, 0.00075% (w/v) LMNG, 0.00025% (w/v) GDN, 0.00015% (w/v) CHS, 10 μM 6-OAU and 100 μM TECP. Peak fractions of the complex were pooled and concentrated to 20 mg/ml for cryo-EM studies.

### Cryo-EM sample preparation and data acquisition

For cryo-EM grid preparation of the 6-OAU-GPR84-Gi complex, 3 μl of the purified complex at 20 mg/ml was applied onto a glow-discharged holey carbon grid (Quantifoil, Au200 R1.2/1.3). Grid was plunge-frozen in liquid ethane using Vitrobot Mark IV (Thermo Fischer Scientific). Cryo-EM imaging was performed on a Titan Krios electron microscope at 300 kV accelerating voltage using a Gatan K3 Summit direct electron detector with an energy filter. Micrographs were collected with a nominal magnification of 81,000× using the EPU software in super-resolution mode with a calibrated pixel size of 0.535 Å and a defocus range of −1.2 to −2.2 μm. Each stack was acquired with an exposure time of 3.5 s and dose-fractionated to 32 frames with a total dose of 55 e^−^Å^−2^. A total of 5307 movies were collected for 6-OAU-GPR84-Gi complex.

### Data processing, 3D reconstruction and modeling building

Image stacks were subjected to beam-induced motion correction using MotionCor2^73^. Contrast transfer function (CTF) parameters were estimated from motion-corrected images using Gctf^74^. Total of 8,056,512 particles of 6-OAU-GPR84-Gi complex were auto-picked using RELION 3.1^75^ and then subjected to reference-free 2D classification to discard poorly defined particles. After several rounds of 3D classification, two well-defined subsets with 628,450 particles were selected. Further 3D classification focusing the alignment on the receptor and complex, produced three high-quality subsets accounting for 62,864 particles. These particles were subsequently subjected to 3D refinement, CTF refinement, and Bayesian polishing, which generated a map with an indicated global resolution of 3.0 Å at a Fourier shell correlation (FSC) of 0.143.

The Alphafold predicted structure of GPR84 was used as initial model for model rebuilding and refinement against the electron microscopy map. The model was docked into the electron microscopy density map using Chimera^76^ followed by iterative manual adjustment and rebuilding in COOT^77^. Real space refinement and rosetta refinement were performed using Phenix programs^78^. The model statistics was validated using MolProbity^79^. Structural figures were prepared in Chimera and PyMOL (https://pymol.org/2/). The final refinement statistics are provided in Supplementary Table 1. The extent of any model overfitting during refinement was measured by refining the final model against one of the half-maps and by comparing the resulting map versus model FSC curves with the two half-maps and the full model. Surface coloring of the density map was performed using UCSF Chimera^76^.

### Molecular dynamics simulation and molecular docking

After removing the G_i_ protein from the 6-OAU-GPR84-G_i_ cryo-EM structure obtained in this study, the 6-OAU-GPR84 complex was subjected to molecular dynamics (MD) simulations in apo and holo states in protein-lipid-water-ions environment. Membrane was modelled in a 1:1 molar ratio of DOPC:POPC. CHARMM-GUI was used to assemble the simulation systems^80^. In the holo simulations, D66 was modelled in its protonated state, as reported for A-type GPCRs^81^; while in the apo simulations, D66 was modelled in its deprotonated state together with a sodium ion. The missing ICL3 (~100 missing residues) was modelled as a 16-residue loop made by joining the first 8 and last 8 residues of the missing ICL3 and constructed using Modeller by building 10000 models. DOPE score was used to choose the best model^82^. Besides 6-OAU, the presence of a cholesterol (CLR) molecule that binds to TM2–4 in the cryo-EM structure was also taken into account in MD simulations (with and without CLR). The CLR binding site in the cryo-EM structure is similar to the CLR-binding site seen in β2-adrenergic receptor, which is proposed to allosterically modulate ligand binding at the orthosteric site^83^. Nonetheless, in our simulations, we find that CLR unbinds from GPR84 and remains unbound; Moreover, the presence of CLR has no notable influence on the ligand and protein dynamics in the apo and holo simulations, respectively.

Four replicas were simulated for each of the following four systems: apo-CLR, apo-noCLR, holo-CLR and holo-noCLR. Each replica was simulated for *ca*. 1.25 μs, totalling *ca*. 20 μs of simulations across all states. The simulation systems comprised *ca*. 75,000 atoms. CHARMM36^84^ forcefield was employed for the MD simulations. The systems were first subjected to an energy minimization for 10,000 steps and followed by gradual heating from 0 to 310 K for 500 ps, using a Langevin thermostat with heavy atoms restrained at 10 kcal mol^−1^ Å^−2^ in an NVT ensemble. The heated systems were subjected to eight successive rounds of 1 ns equilibration steps. During the equilibration, protein and ligand heavy atoms were subjected to harmonic restraints, and lipids were subjected to planar restraints to maintain bilayer planarity. The harmonic restraints for each step were relaxed progressively going from 10 to 0.1 kcal mol^−1^ Å^−2^. The equilibrations were performed at a 1 fs timestep at *T* = 310 K and *P* = 1 bar using the Langevin thermostat and Nosé–Hoover Langevin barostat in NPT ensemble. The production runs were performed with a hydrogen mass repartitioning scheme with a timestep of 4.0 fs with a nonbonded cutoff at 12 Å^85^. Long-range electrostatics were evaluated with the Particle Mesh Ewald (PME) method. Protein and lipid bond lengths were constrained with the SHAKE algorithm. NAMD 2.14 was used for MD simulations^86^. Glide was used to perform the molecular docking^87^.

### GTPγS binding assay

Studies on the potency of 6-OAU to activate GPR84 and how this was altered by variation at specific residues were conducted using a series of GPR84-Gα_i2_ fusion protein^49,88^. Point mutation of residues predicted from the structural data to modify binding and or function of 6-OAU were introduced into such fusion proteins and expressed either stably in Flp-In T-REx 293 cells or transiently into HEK293T cells. The ability of varying concentration of 6-OAU to promote binding of [^35^S] GTPγS was then assessed as in our previous studies^50^. Briefly, membrane fractions of Flp-In T-REx 293 or HEK293T cells were incubated in buffer containing 20 mM Hepes pH 7.5, 5 mM MgCl_2_, 160 mM NaCl, 0.05% fatty-acid-free bovine serum albumin, and various concentrations of ligands. Then, [^35^S] GTPγS (50 nCi per reaction) with 1 μM GDP was added and the mixture was incubated at 30°C for 1h. The reaction was terminated by adding cold PBS buffer and the membrane fractions were collected by rapid vacuum filtration through GF/C glass fiber prefilters using a UniFilter FilterMate Harvester (PerkinElmer). After three additional washes with cold PBS, the filters were dried and incubated with MicroScint-20 (PerkinElmer). [^35^S] GTPγS binding to G_i_ was quantified by liquid scintillation spectroscopy. The data were analyzed by GraphPad Prism 6 (GraphPad Software).

## Data Availability

The 3D cryo-EM density map of 6-OAU-GPR84-G_i_ has been deposited in the Electron Microscopy Data Bank under the accession numbers EMD-29645. Atomic coordinates for the atomic model have been deposited in the Protein Data Bank under the accession numbers 8G05.
